# Maternal high-fat diet consumption induces sex-dependent alterations of the endocannabinoid system and redox homeostasis in liver of adult rat offspring

**DOI:** 10.1038/s41598-018-32906-0

**Published:** 2018-10-03

**Authors:** Rosiane Aparecida Miranda, Mariana Macedo De Almeida, Camilla Pereira Dias Da Rocha, Larissa de Brito Fassarella, Luana Lopes De Souza, Aline Fonseca Pereira De Souza, Cherley Borba Vieira De Andrade, Rodrigo Soares Fortunato, Carmen Cabanelas Pazos-Moura, Isis Hara Trevenzoli

**Affiliations:** Carlos Chagas Filho Biophysics Institute, Federal University of Rio de Janeiro, Rio de Janeiro, Brazil

## Abstract

Maternal diet plays a critical role in health development. Perinatal overnutrition induces metabolic dysfunctions and obesity in the offspring. Obesity is associated with endocannabinoid system (ECS) over activation and oxidative stress. Liver ECS activation induces hepatic steatosis, inflammation and fibrosis while the antagonism of cannabinoid receptors ameliorates these alterations. Here, we investigated the effect of perinatal maternal high-fat diet (HF, 29% of calories as fat) on the ECS and antioxidant system in liver of male and female adult rat offspring (180 days old). Maternal HF diet increased hepatic cannabinoid receptors, ECS metabolizing enzymes and triglyceride content, with male offspring more affected. ECS changes are likely independent of estradiol serum levels but associated with increased hepatic content of estrogen receptor, which can stimulate the expression of ECS components. Differently, maternal HF diet decreased the activity of the antioxidant enzymes glutathione peroxidase, superoxide dismutase and catalase, and increased oxidative stress markers in both sexes. Alterations in the redox homeostasis were associated with mitochondria damage but not with liver fibrosis. Our data suggest that maternal HF diet induces ECS over activation in adulthood, and that male offspring are at higher risk to develop liver disease compared with female rats.

## Introduction

Environmental conditions during critical developmental periods such as gestation and lactation predict the lifelong health of the offspring, a phenomenon known as “programming”^1^. Maternal exposure to under or overnutrition, stress, endocrine disruptors and other types of insults may program metabolic dysfunctions in the offspring including diabetes, cardiovascular diseases, dyslipidemia and obesity^2,3^. We have shown that maternal high-fat (HF) diet during gestation and lactation induces obesity in the offspring associated with thyroid and adrenal dysfunction, hyperleptinemia with hypothalamic leptin resistance, hepatic dysfunction, dyslipidemia, hyperphagia and increased appetite for fat across different periods of life^4–8^.

Obesity is an important risk factor for liver dysfunctions such as non-alcoholic fatty liver disease (NAFLD)^9^. We previously demonstrated that maternal HF diet increased hepatic triglycerides, total lipid content, free cholesterol in parallel to reduced cholesterol ester and higher liver weight of adolescent rat offspring, which could predispose to later NAFLD^5^.

Obesity and NAFLD are associated with dysfunctions in the endocannabinoid system (ECS) in humans and animal models^10,11^. ECS comprises the main endogenous cannabinoids (ECs), anandamide (AEA) and 2-aracdonoylglycerol (2-AG), which are produced from arachidonic acid from membrane phospholipids. ECs regulate energy and lipid metabolism through central and peripheral mechanisms via cannabinoid receptors subtypes 1 and 2 (CB1 and CB2), and ECs are degraded by fatty acid amide hydroxylase (FAAH) and monoacylglycerol lipase (MAGL)^11,12^.

CB1 and CB2 are expressed in the central nervous system (CNS), heart, uterus, intestine, brown and white adipose tissue, cells of the immune system and liver^13–15^. In the liver, ECs increase lipid accumulation and reactive oxygen species (ROS)^16^. CB1 activation increases oxidative stress and decreases antioxidant activity, whereas CB2 receptors are related to the suppression of ROS generation in different tissues^17^. On the other hand, CB1 antagonism improves liver steatosis, decreases serum triglyceride levels and increases serum high-density lipoprotein (HDL)^18,19^.

Hepatic diseases are also associated with the accumulation of cellular lesions caused by deregulation of redox homeostasis, which may result from increased ROS, such as superoxide and hydrogen peroxide (H_2_O_2_), or decreased antioxidant defense of the enzymes superoxide dismutase (SOD), glutathione peroxidase (GPx) and catalase (Cat)^20^. The disarrangement of this system leads to cellular oxidative stress, with decreased total thiols and increased residues of carbonylated proteins^21^ and derivatives from lipid peroxidation like 4-hydroxynonenal (4-HNE)^22^.

Although studies have demonstrated a relationship between ECS and the cellular redox homeostasis in liver^22,23^, the interconnection between ECS and oxidative stress in metabolic programming is mostly unknown. We hypothesized that maternal HF diet consumption during perinatal phase would program hepatic dysfunctions associated with dysregulation of ECS and redox homeostasis in the liver of adult rat offspring. Because ECS is known to be regulated by sex steroids^24,25^, we further hypothesized that liver ECS programming would occur in a sex dependent manner and liver ECS would be differentially regulated by estradiol levels in female adult rats.

## Results

Maternal HF diet increased body weight in male and female adult offspring at 180 days old (p = 0.017, p = 0.014, respectively) as well as subcutaneous fat pad mass (p = 0.025; p = 0.001, respectively) (Table 1). Besides, maternal HF diet increased the brown adipose tissue mass, and serum levels of glucose and triglycerides (p = 0.01; p = 0.01; p = 0.04, respectively) in male adult offspring, without differences in females. In female adult offspring from HF dams, we observed diminished estradiol levels (p = 0.0007), without differences in males. No differences were observed in aspartate aminotransferase (AST) and alanine aminotransferase (ALT) in male or female adult offspring (Table 1).

**Table 1:** Effect of maternal obesity on biometrical and biochemical parameters in both male and female offspring at 180-day-old.

Parameters	Males		*p* value	Females		*p* value
	C	HF		C	HF	
Body weight (g)	525.4 ± 13.5	558.9 ± 11.5^*^	0.017	306.0 ± 3.37	316.2 ± 3.27^*^	0.014
Visceral fat pad (g)	7.78 ± 0.74	9.42 ± 0.60	0.095	2.85 ± 0.12	3.190 ± 0.16	0.105
Subcutaneous fat pad (g)	10.4 ± 0.80	13.9 ± 0.65^**^	0.025	3.98 ± 0.15	5.17 ± 0.34^**^	0.001
Brown fat pad (g)	0.50 ± 0.01	0.61 ± 0.03^*^	0.014	0.29 ± 0.01	0.32 ± 0.01	0.241
Liver weight (g)	16.9 ± 0.77	18.1 ± 0.56	0.214	10.7 ± 0.37	10.9 ± 0.27	0.832
Glucose (mg/dL)	158.8 ± 8.5	201.5 ± 12.3^*^	0.010	169.7 ± 8.3	155.7 ± 7.6	0.235
Insulin (µIU/mL)	49.26 ± 3.3	51.96 ± 3.1	0.564	42.36 ± 5.1	51.13 ± 5.1	0.241
Triglycerides (mg/dL)	123.1 ± 14.4	168.1 ± 14.6^*^	0.044	89.71 ± 18.0	115.6 ± 10.3	0.227
Estradiol (pg/mL)	96.41 ± 5.4	89.98 ± 5.6	0.424	111.3 ± 5.3	79.9 ± 5.4^***^	0.0007
AST (U/L)	164.8 ± 15.5	166.9 ± 13.0	0.918	211.8 ± 18.3	181.1 ± 13.7	0.191
ALT (U/L)	75.45 ± 6.2	62.58 ± 3.4	0.085	72.36 ± 4.8	100.3 ± 13.5	0.066

The data represent the mean ± SEM obtained from 10–15 rats per experimental group. The symbols represent significant differences by Student’s *t* test. AST: aspartate aminotransferase; ALT: alanine aminotransferase.

**Figure 1:**
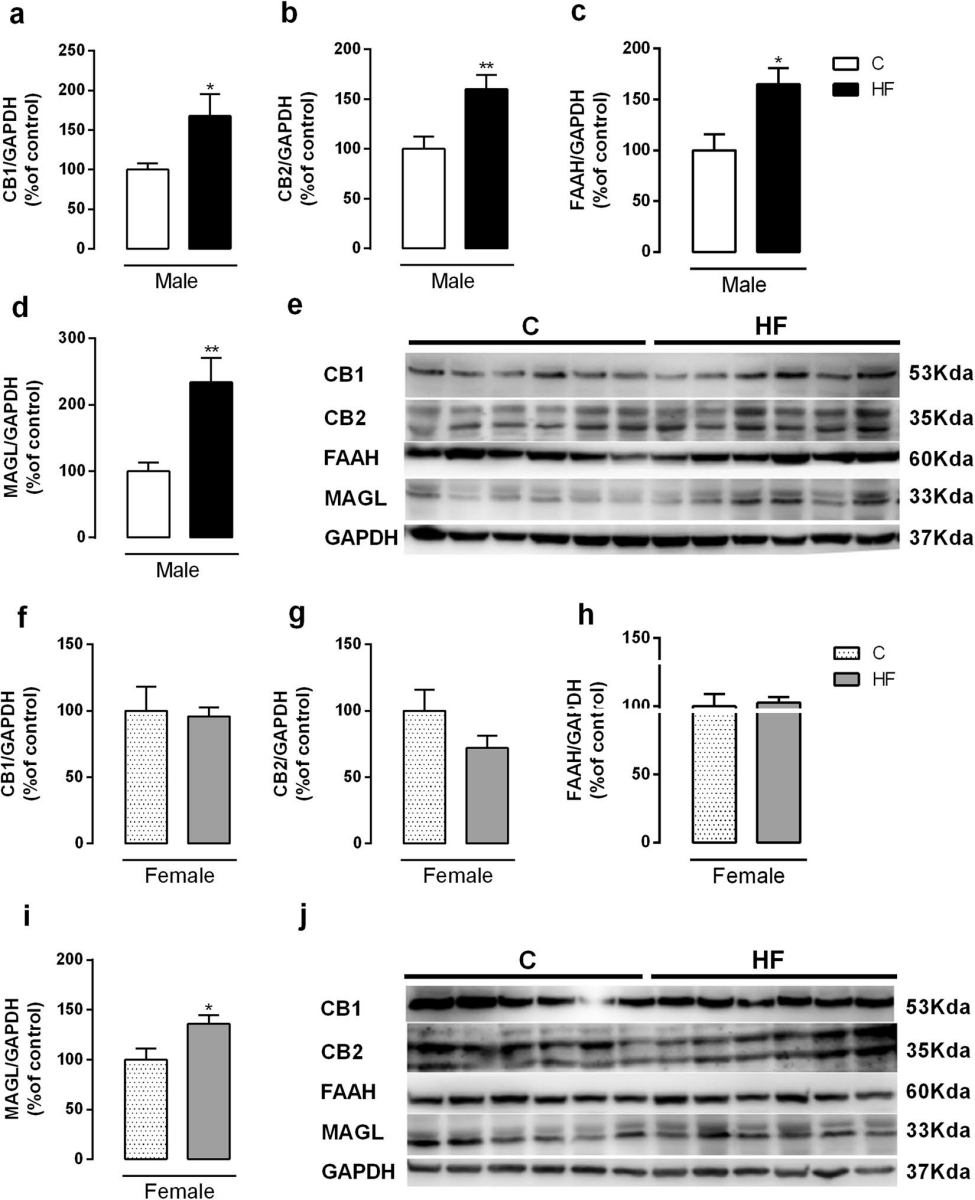
Effect of maternal HF diet on hepatic protein expression of CB1 (**a**,**f**), CB2 (**b**,**g**), FAAH (**c**,**h**), MAGL (**d**,**i**) in both male and female offspring at 180-day-old. Representative western blot bands for each protein were showed (**e**,**j**). Data is expressed as mean ± S.E.M, n = 6–7 per group; *p < 0.05, **p < 0.001, by Student’s *t* test.

In the liver of male offspring, maternal HF diet increased CB1 and CB2 protein content (p = 0.02; p = 0.008, respectively) (Fig. 1a,b**)**, FAAH content (p = 0.01) (Fig. 1c) and MAGL content (p = 0.003) (Fig. 1d). However, female HF offspring only presented increase in MAGL (p = 0.03) (Fig. 1i).

**Figure 2:**
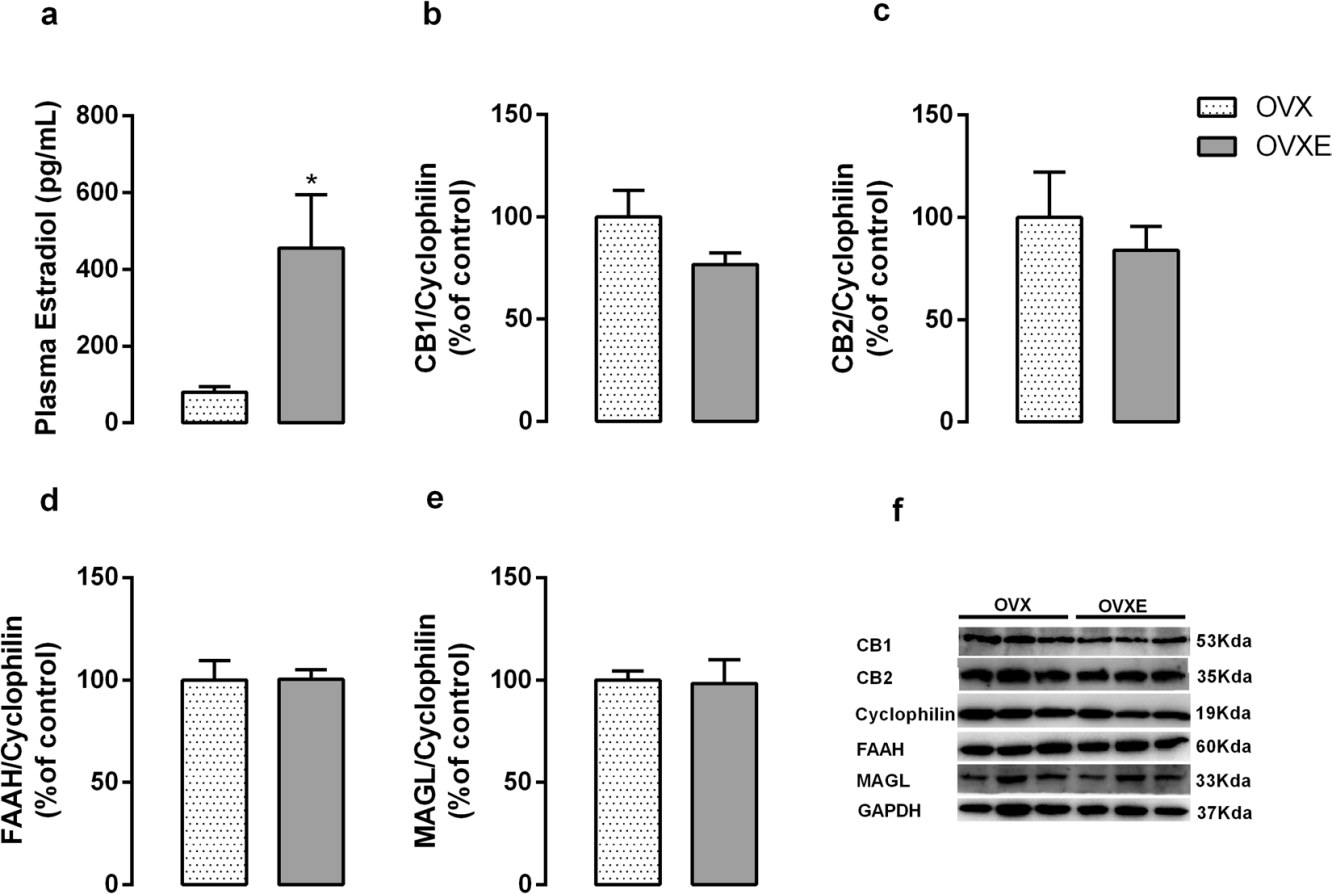
Effect of ovariectomy and estradiol treatment on estradiol levels (**a**) and hepatic protein expression of CB1 (**b**), CB2 (**c**), FAAH (**d**) and MAGL (**e**) of adult rats. Representative western blot bands for each protein were showed (**f**). Data is expressed as mean ± S.E.M, n = 6–9 per group; *p < 0.05, by Student’s *t* test.

Because we found reduced plasma estrogen levels in the female HF offspring, we evaluated the influence of estradiol circulating levels on the liver ECS components. We performed ovariectomy (OVX) and estradiol treatment in OVX female control rats (OVXE). The estradiol levels were higher in OVXE (p = 0.04) compared with OVX rats (Fig. 2a), as expected. However, we did not observe alterations in liver ECS between OVX and OVXE rats (Fig. 2b-e).

**Figure 3:**
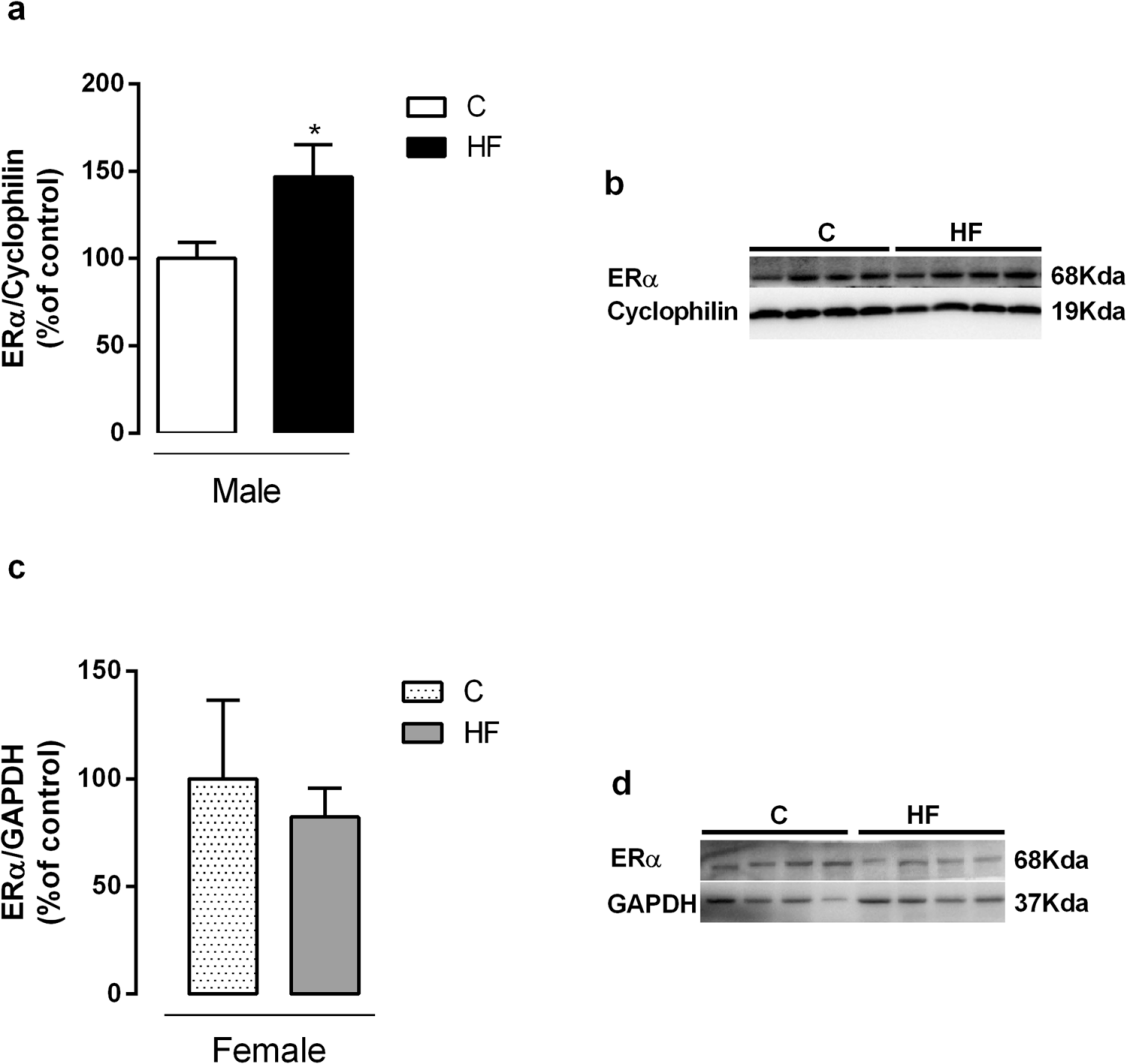
Effect of maternal HF diet on Estrogen receptor alpha (ERα) in liver in both male (**a**) and female (**c**) offspring at 180-day-old. Representative western blot bands for each protein were showed (**b**,**d**). Data is expressed as mean ± S.E.M, n = 7 per group, *p < 0.05, by Student’s *t* test.

We also evaluated the protein expression of ERα in liver of C and HF offspring, and the ERα expression was higher in HF male offspring (p = 0.04) compared with sex-matched controls, without changes in female offspring (Fig. 3a,b).

**Figure 4:**
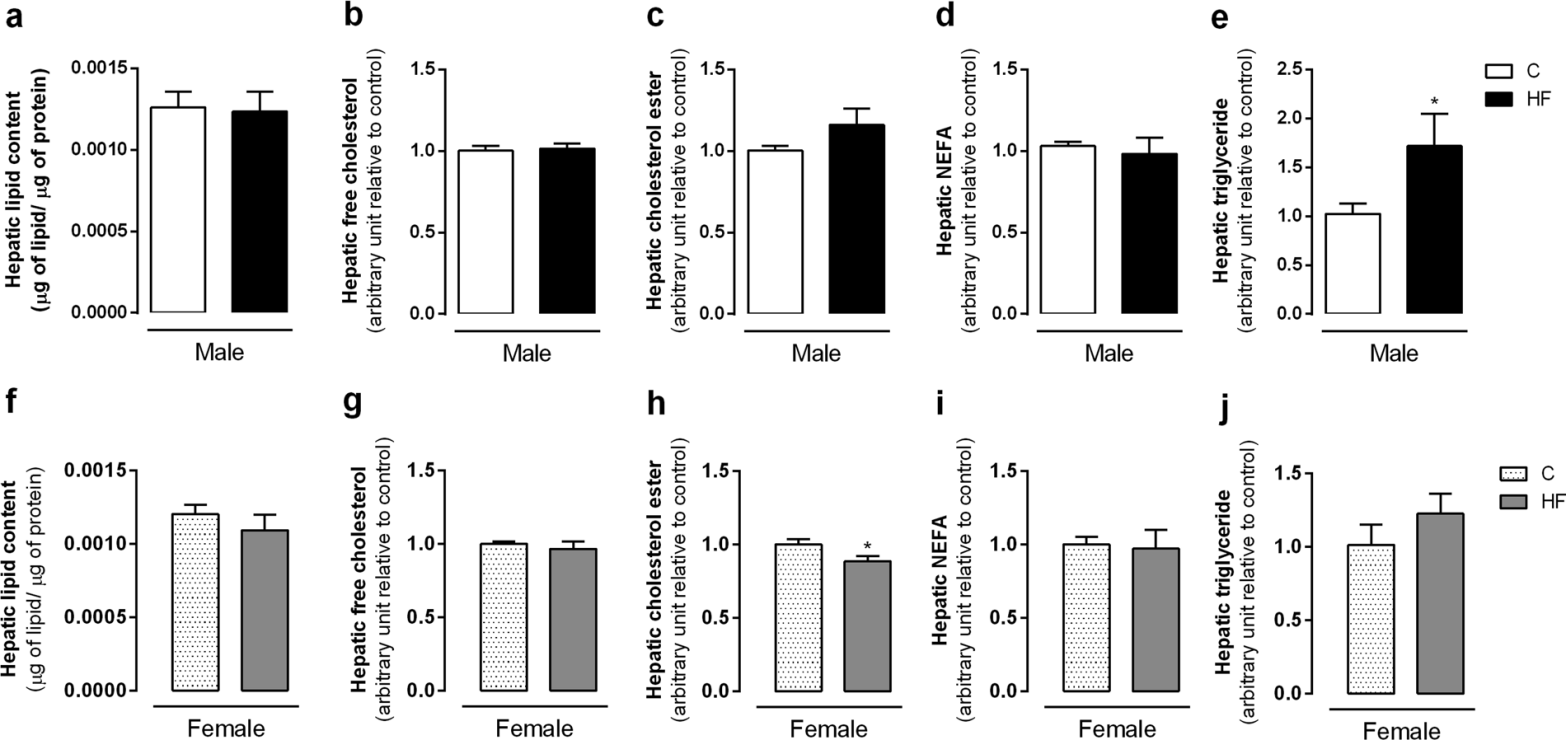
Effect of maternal HF diet on hepatic lipid content (**a**,**f**), free cholesterol (**b**,**g**), cholesterol ester (**c**,**h**), NEFA (**d**,**j**), triglyceride (**e**,**j**) in both male and female offspring at 180-day-old. Data is expressed as mean ± S.E.M, n = 9–12 per group; *p < 0.05, by Student’s *t* test.

ECS changes in the liver occurred in parallel to increased hepatic triglycerides content (p = 0.04) only in male offspring (Fig. 4e). Maternal HF diet did not change hepatic total lipid content, free cholesterol, cholesterol ester and NEFA (Fig. 4a-j) in male or female offspring.

**Figure 5:**
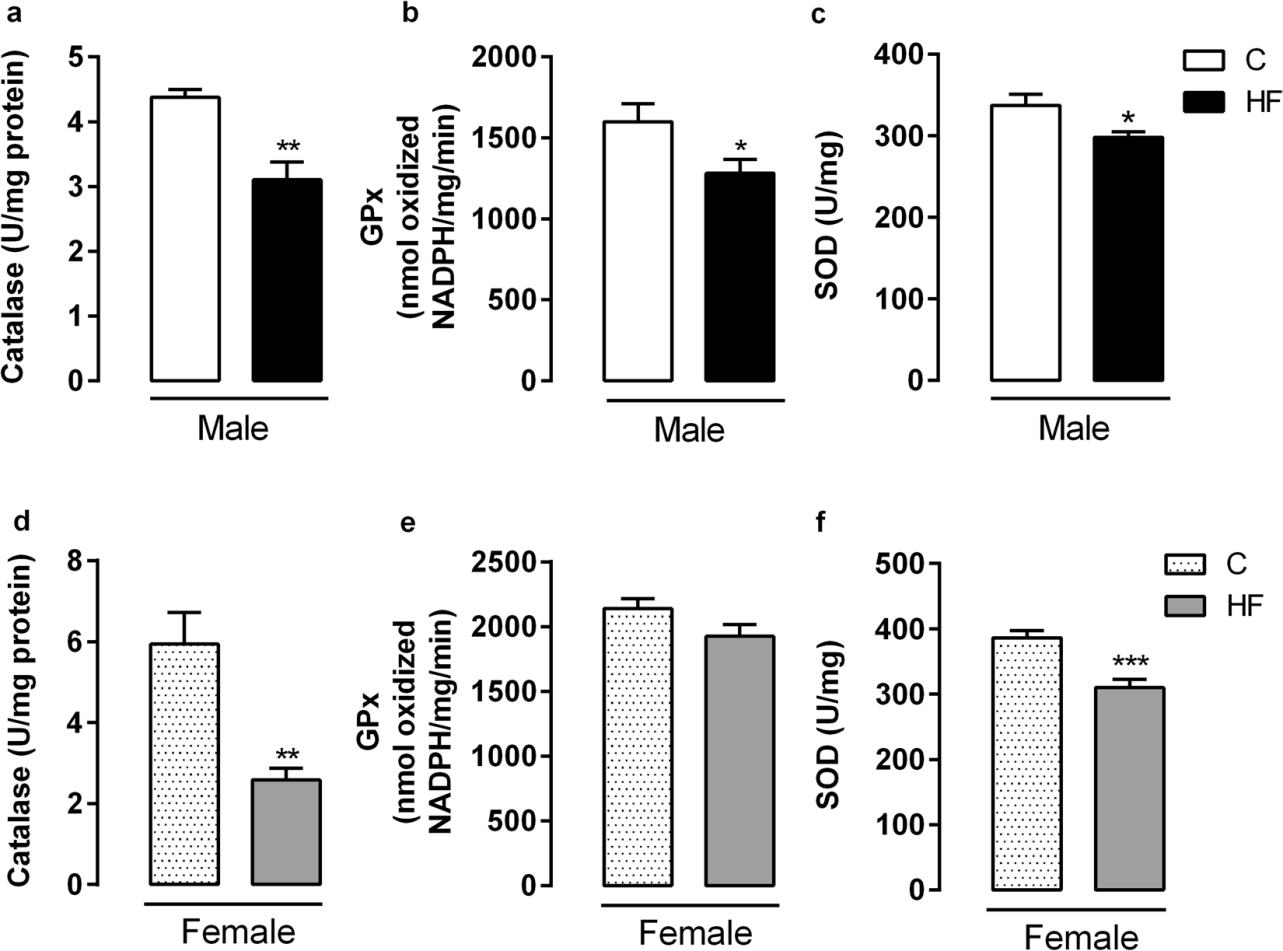
Effect of maternal HF diet on hepatic activity of antioxidant enzymes, catalase (**a**,**d**), glutathione peroxidase (GPx) (**b**,**e**), superoxide dismutase (SOD) (**c**,**f**) in both male and female offspring at 180-day-old. Data is expressed as mean ± S.E.M, n = 8 per group; *p < 0.05, **p < 0.001, ***p < 0.0001, by Student’s *t* test.

**Figure 6:**
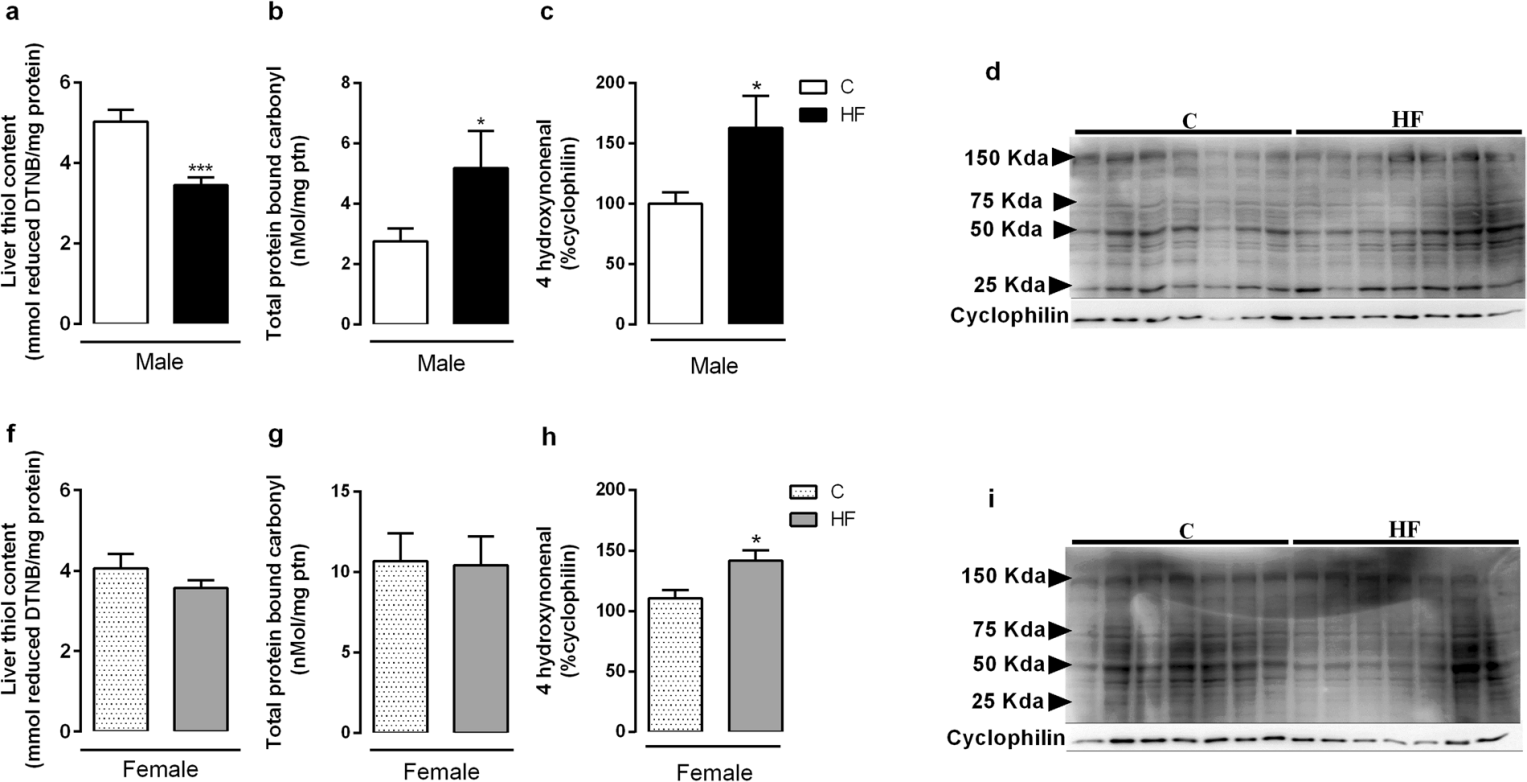
Effect of maternal HF diet on hepatic biomarkers of oxidative stress: thiol content (**a**,**f**), total protein bound carbonyl (**b**,**g**) and 4-HNE (**c**,**h**) in both male and female offspring at 180-day-old. Representative western blot bands for each protein were showed (**d**,**i**). Data is expressed as mean ± S.E.M., n = 7–8 per group; *p < 0.05, ***p < 0.0001, by Student’s *t* test.

We also evaluated the main enzymes of antioxidant system in the liver of adult offspring. Maternal HF diet decreased Cat activity (Fig. 5a,d) (p = 0.001), and SOD activity (Figs. 5c,f) both in male and female offspring (p = 0.04; p = 0.0006, respectively), while it decreased GPx activity (p = 0.04) only in male rats (Fig. 5b). Regarding the oxidative stress biomarkers, maternal HF diet decreased liver thiol content (p = 0.0007) (Fig. 6a), and increased protein carbonyl and 4-HNE content (p = 0.04; p = 0.03) in male rats (Fig. 6b,c). In female rats, maternal HF diet only increased the 4-HNE content (p = 0.02) (Fig. 6h). However, maternal HF diet did not change the H_2_O_2_ generation in membrane or microsomal fraction in both male and female offspring.

**Figure 7:**
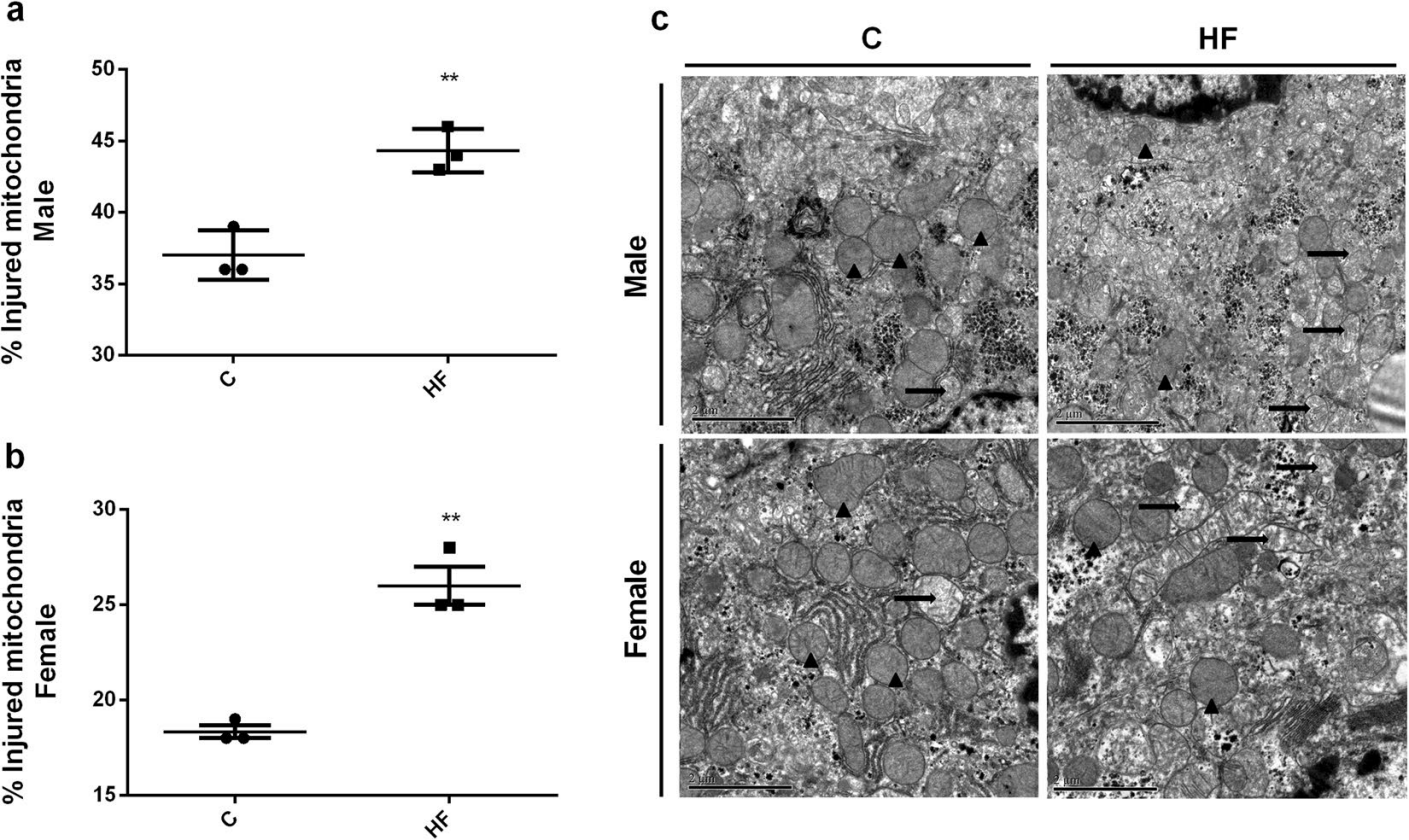
Effect of maternal HF diet on percentage of injured mitochondria in liver in both male (**a**) and female (**b**) offspring at 180-day-old. Representative images are shown (**c**). Data is expressed as mean ± S.E.M, n = 3 per group; **p < 0.001, by Student’s *t* test.

We quantified the percentage of injured mitochondria by Transmission Electron Microscopy and we observed that maternal HF diet was able to induce injury in both male and female offspring (p = 0.005; p = 0.001, respectively) (Fig. 7a,b**)**.

**Figure 8:**
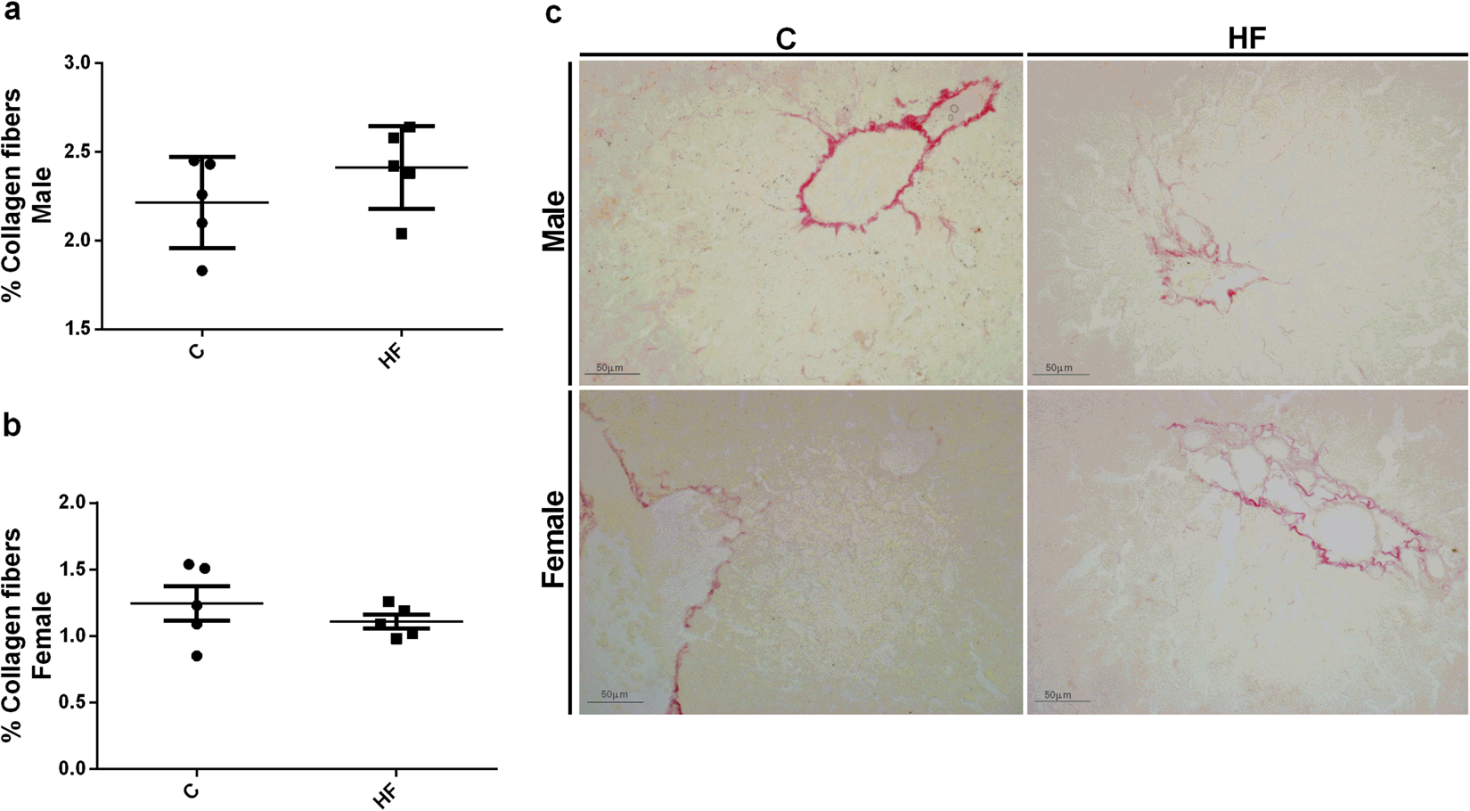
Effect of maternal HF diet on percentage of collagen fibers in liver in both male (**a**) and female (**b**) offspring at 180-day-old. Representative images are shown (**c**). Data is expressed as mean ± S.E.M, n = 5 per group; *p < 0.05, by Student’s *t* test.

To evaluate the impact of ECS and redox homeostasis changes on liver morphology, we performed the picrosirius red staining of collagen fibers to investigate fibrosis. However, we observed no effect of maternal HF diet on collagen fibers content in male or female offspring (Fig. 8).

## Discussion

In the current study, we investigated the ECS in liver of the male and female adult rat offspring from HF fed dams in parallel to the prooxidant and antioxidant profile. We observed that male HF offspring phenotype at adulthood corroborated previously data published by our group^6,8^ and others^26^. Male and female offspring from HF dams displayed higher body weight and adiposity. This phenotype is observed already at weaning in this model, which is associated with changes in the milk composition from HF fed dams with higher lipid and protein content^4^.

The main finding of the present study is that maternal HF diet during perinatal life induced sex-specific long-term changes in the liver ECS components of the adult offspring. Maternal HF diet increased protein expression of CB1 and CB2 receptor in male offspring. It has been shown that upregulation of CB1 in liver is related with hepatic fibrosis, steatosis and lipid accumulation in humans, animal models and hepatocytes *in vitro*^27–29^, and on the other side, the blockage of CB1 receptor reverses these dysfunctions^28^.

Different of CB1, the role of CB2 receptor on metabolic parameters is controversial or dual^30^. In obese children with NAFLD, a polymorphism of the CB2 gene (*Cnr2)* is associated with severity of liver inflammation and inflammatory steatohepatitis (NASH)^31^. In obese women, CB2 mRNA levels in liver correlates positively with hepatic Acetyl-coenzyme A carboxylase 1 (ACC1) gene expression, a key lipogenic enzyme of the *de novo* synthesis of fatty acids^32^. Besides, it is associated with increased PPARγ, IL6, TNFα, resistin, suggesting a deleterious role promoting liver lipid accumulation and inflammation. Paradoxically, CB2 mRNA levels were also positively correlated with the levels of the adipocyte-derived hormone adiponectin in the liver^32^, which presents beneficial effect on steatosis, insulin sensitivity, oxidative stress and inflammation^33^. In cirrhotic human liver, CB2 activation develops an antifibrogenic role during chronic liver injury^34^. On the other hand, in *Cnr2* −/− HFD-fed mice model, the absence of CB2 receptors decreases the steatosis and liver triglyceride concentration caused by HF diet, suggesting that CB2 activation contributes to liver inflammation and hepatic steatosis^35^. Thus, increased hepatic CB1 and CB2 in HF male offspring may represent a higher risk to develop liver steatosis and fibrosis.

Increased triglyceride hepatic content indicates liver injury probably induced by HF diet^36–38^. In nonhuman primate model, fetuses from obese mothers fed HF diet have increased liver triglycerides content^39^, suggesting that developing fetuses of different species are highly vulnerable to excess of lipids in maternal diet. In fact, the analysis of lipid profile showed that HF male offspring had increased hepatic content of triglycerides and hipertrigliceridemia. However, maternal HF diet did not affect serum levels of AST and ALT neither the amount of collagen fibers in the liver, which are markers of severe hepatic damage. Thus, we speculate that the profile found in liver ECS of the offspring may contribute to a more drastic metabolic phenotype under a second nutritional hit, such as a high-fat diet challenge in adulthood, as previously demonstrated by other studies^40,41^.

Interestingly, we observed a marked sex-specific regulation of the hepatic ECS by maternal HF diet. Sex-specific programming is an interesting topic and it has been demonstrated in several experimental models, including the maternal HF diet rat model^42,43^. However, very little is known regarding the direct ECS regulation by sex steroids, even in control rats, mice or humans^24^. In our previous studies^7,8^, we characterized that the ECS is differentially regulated in male and female HF offspring in other tissues, such as white and brown adipose tissue and hypothalamus. Interestingly, ECS changes can occur even before the major physiological alterations in sex hormone levels during puberty, since we observed that maternal HFD induces ECS sex-specific changes already at birth^8^ and also at weaning^7^. In the present study, maternal HF diet increased the ECS expression in liver of adult male offspring with minor changes in females. In adulthood, ECS changes occurred in parallel to decreased serum levels of estradiol in female offspring. To test whether this serum alteration was associated with the sex-dependent ECS changes, we developed the OVX model. However, the modulation of estradiol levels at adulthood did not affect ECS expression in the liver. It is possible that liver ECS changes induced by maternal HF diet occurred before the age analyzed in the present study, similar to the profile we observed in other peripheral tissues and may not be directly associated with changes in serum estradiol. A possible mechanism would be the modulation of estrogen receptor (ER) expression in the liver, rather than estradiol serum levels. It has been reported the presence of response elements to estrogen in *Cnr1* and *Faah* promoter region^44,45^, and estrogen has a stimulatory effect on ECS components^25^. Here, we showed that maternal HF diet increased the ER levels in liver of male offspring but not in female offspring, which may contribute to the increased CB1, CB2 and FAAH in males.

To our knowledge, only two studies have been conducted to characterize the effect of maternal HF diet on hepatic expression of the ECS components. The first one conducted by Ramirez *et al*., showed that maternal hypercaloric-hypoproteic palatable diet decreases mRNA levels of FAAH and MAGL in the liver of female adult rat offspring^46^, rather than changes in male offspring as we showed here. Although interesting, these results cannot be directly compared with the present study because there are relevant differences in the offspring phenotype, such as decreased offspring body weight. In adult offspring programmed by perinatal undernutrition, it was demonstrated decreased hepatic mRNA levels of CB1, FAAH and MAGL only in female offspring^47^. However, of note, the present study focused on the regulation of the protein content of ECS components instead of transcript levels. Another study recently published by Gandhi *et al*., showed that maternal HF diet in baboons decreases CB2 and FAAH protein content in fetal liver, but the impact of ECS changes on liver morphology and function was not investigated^48^. The different profiles found in liver ECS among these studies may arise from different ages and species used in the experiments.

In this study, we showed that maternal HF diet increased FAAH and MAGL protein expression in the liver of offspring. MAGL inhibition suppresses oxidative stress and inflammation in the liver^49^, and FAAH inhibitor decreases ROS production and improves hepatic antioxidant response^22^. Thus, the increased ECS enzymes may contribute to impaired redox homeostasis in the liver of HF offspring. These findings suggest an interplay of ECS and redox system and may be important components of study for therapeutic targets in metabolic diseases.

Liver injury is frequently associated with oxidative stress, which is characterized by an imbalance between prooxidant and antioxidant systems resulting in increased intracellular ROS availability^50^. We observed decreased activity of the antioxidant enzymes Cat, GPx and SOD in both male and female HF offspring, suggesting a deleterious impact of maternal HF diet on antioxidant defense. In fact, we also observed alterations in markers of oxidative stress such as decreased thiol levels and increased total protein bound carbonyl and 4-HNE content in HF offspring. These biochemical alterations occurred in parallel to increased mitochondrial damage in the liver.

There are some evidences that ECS can modulate ROS production by alterations in the activity of prooxidant and antioxidant enzymes. Jia, *et al*.^51^ demonstrated that treatment of neuronal cells with AEA led to decreased intracellular ROS and NADPH oxidase 2 (Nox2), which were reversed by the CB1 antagonist. They also demonstrated that under oxidative stress, AEA increases SOD and GPx activity, suggesting improvement of the antioxidant defense. In mice presenting NAFLD, CB1 antagonism improves liver oxidative stress^52^. Moreover, CB2 agonists protect against hepatic injury induced by ischemia and decrease tissue free radical damage, suggesting that CB2 activation could be a protective pathway^53,54^.

Although maternal HF diet induced minor changes in the liver ECS of female offspring, it induced significant changes in the antioxidant profile and oxidative stress markers in both male and female adult rats. These findings suggest that alteration in additional pathways, independent on the ECS, contribute to the oxidative balance in liver of female offspring.

We consider as main limitation of the present study the lack of measurement of the tissue levels of the major endocannabinoids AEA and 2-AG in the offspring liver. In addition, a more detailed analysis of molecular and morphological markers of liver injury would provide a better picture of the impact of maternal HF diet on liver metabolism and inflammatory status.

In conclusion, we demonstrated that maternal HF diet induced overweight, increased adiposity, dyslipidemia, over expression of the ECS and impairment of the antioxidant capacity in liver of adult offspring, with male rats more affected. Of note, these alterations did not impact the levels of liver fibrosis or serum hepatic enzymes, which could possibly be triggered by second hits such as postnatal exposure to obesogenic factors. The present data is a contribution to characterize the impact of maternal HF diet on liver ECS in parallel to oxidative stress markers in the offspring, and the comprehension of these pathways may contribute to insights into new pharmacological targets for treatment of liver disease, considering sex differences in the liver ECS regulation.

## Methods

### Animals

The handling of the experimental animals followed the American Physiological Society’s guiding principles^55^ and was previously approved by the Animal Care and Use Committee of the Carlos Chagas Filho Biophysics Institute (process number 123/14 and 089/17). For the experimental programming model induced by maternal HF diet consumption, 60-day-old female Wistar rats (180–220 g) were obtained from the Center of Rat Reproduction of the Federal University of Rio de Janeiro, Rio de Janeiro, Brazil. Progenitor rats received control or experimental diets as detailed in the section “experimental groups and diets”.

A separate subset of female rats underwent a bilateral ovariectomy (OVX) to investigate the role of modulation of the estradiol serum levels on ECS components in the liver at adulthood. Rats were anesthetized with ketamine/xylazine (30/6 mg/kg, intramuscular). A midline incision was made to expose the lower abdominal cavity. Rats were randomly divided into 2 groups: OVX and OVX treated with estradiol (OVXE). In the OVXE group, the rats were administered with 7 *µ*g/kg/day of β-estradiol-3-benzoate (Sigma-Aldrich, USA) by subcutaneous injection for 7 days beginning 10 days after surgery, while group OVX received vehicle (soy oil) in the same volume. We evaluated the ECS in liver samples from both groups by western blotting.

For all animal procedures, rats were housed under controlled conditions in a 12-h light-dark cycle (lights on from 7 a.m. to 7 p.m.) and at room temperature of 23 ± 2 °C. Water and diets were offered *ad libitum*.

### Experimental groups and diets

Female progenitor rats were randomly chosen and divided in two groups: control group (C) which received a standard diet (9% of the calories as fat), and a high-fat group (HF) which received a HF diet (28.6% of the calories as fat) as previously described by our group^4–8^. Progenitor rats received the diets during 8 weeks before mating and throughout gestation (21 days) and lactation (21 days). After mating, pregnant rats were housed in individual standard rat cages until delivery. At birth, the litters were adjusted to three male and three female pups per each dam, a number that maximizes lactation performance^56^. All pups were weaned at 21-day-old and fed a control chow diet until adulthood. Body weight was recorded weekly from weaning to 180-day-old, when offspring were euthanized to collect and weigh the retroperitoneal and subcutaneous fat pads, liver, and blood samples. For each experimental procedure, except for electronic microscopy, at least 6 animals from different litters were used for each group to avoid litter effects on the statistical analysis.

### Plasma analyses

Blood samples were centrifuged (1233 × g for 15 min, 4 °C) for plasma separation. Glucose, triglycerides, AST and ALT concentration were determined using commercial kits (Gold Analisa®, Belo Horizonte, MG, Brazil). The AST/ALT intra and interassay variation coefficients were 1.5% and 5.9%/ 2.8% and 5.3%, respectively. The limit of detection was 400 U/L. Plasma insulin concentration was determined by radioimmunoassay (Linco Research, MO, USA). The insulin intra- and interassay variation coefficients were 9.8 and 12.2%, respectively. Plasma estradiol levels were determined using a radioimmunoassay kit (MP Diagnostics, 25 USA) with intra-assay variation of 15,7%.

### Western blotting

Protein content of ECS targets and 4-HNE was determined by western blotting in liver homogenate as previously described with some adaptations^7,8^. Each sample was obtained from a different rat from a different litter (n = 7). Anti-CB1, CB2, FAAH, MAGL and 4-HNE were assayed. Liver samples were collected and snap frozen in liquid nitrogen, followed by lysis (pH 6.4 lysis buffer: 50 mM HEPES, 1 mM MgCl_2,_ 4 10 mM EDTA, 1% Triton X-100, containing a protease inhibitor cocktail (Roche^®^)). After centrifugation, total protein content was determined using a BCA^™^ Protein Assay Kit (Thermo Scientific^®^, Rockford, IL, USA). Total liver protein (15ug) were separated by 12% SDS-PAGE, and then transferred to a polyvinylidene difluoride membrane (Hybond-P, 0.45 µm, PVDF, Amersham Biosciences, BKM, England) using Semi-dry system (Bio-rad laboratories, Hercules, CA, USA). The non-specific binding was blocked for 90 min with 5% BSA with continuous shaking and then membranes were incubated overnight at 4 °C with primary antibodies followed by 90 min incubation with the secondary antibody. The specification of each antibody was described in Table 2. Immunoreacted proteins were visualized by ECL prime kit and Image Quant LAS 4000 equipment followed by densitometric analyses (GE Healthcare, Buckingham, Shire, UK). Glyceraldehyde-3-phosphate dehydrogenase (GAPDH) or cyclophilin was used for normalization.

**Table 2:** Antibodies used for western blotting.

Primary Antibodies			Secondary Antibodies		
Antibody	Distributed by	Dilution	Distributed by	Dilution	Specificity
4-HNE	Abcam, MA, USA	1:1000	Amersham Bioscience, Inc	1:10000	Anti-rabbit
CB1	Cayman MI, USA	1:500	Amersham Bioscience, Inc	1:10000	Anti-rabbit
CB2	Sigma-Aldrich MO, USA	1:1000	Cell Signalling Tecnology MA, USA	1:10000	Anti-mouse
Cyclophilin	Cell Signalling Tecnology MA, USA	1:1000	Amersham Bioscience, Inc	1:10000	Anti-rabbit
ERα	Cell Signalling Tecnology MA, USA	1:500	Invitrogen MA, USA	1:10000	Anti-rabbit
FAAH	Cayman MI, USA	1:200	Amersham Bioscience, Inc	1:10000	Anti-rabbit
GAPDH	Cell Signalling Tecnology MA, USA	1:1000	Amersham Bioscience, Inc	1:10000	Anti-rabbit
MAGL	Santa Cruz Biotecnology MA, USA	1:1000	Amersham Bioscience, Inc	1:10000	Anti-rabbit

4-HNE: 4-Hydroxynonenal; CB1: cannabinoid type-1 receptor; CB2: cannabinoid type-2 receptor; ERα: Estrogen receptor alpha; FAAH: fatty acid amide hydrolase; GAPDH: glyceraldehyde-3-phosphate 5 dehydrogenase; MAGL: monoacylglycerol lipase.

### Antioxidant enzyme activities

Approximately 100 mg of liver from both male and female offspring were homogenized in 5 mM Tris HCl, 0.9% NaCl (pH 7.4) containing protease inhibitor cocktail (Roche Diagnostics, IN, USA). The homogenate was centrifuged at 720 × g for 10 min, 4 °C and the supernatant was used for enzyme activity assays. Total protein content was quantified using a Pierce^TM^ BCA Protein Assay Kit (Thermo Scientific, Rockford, USA). All the enzymatic assays were performed in an UV spectrophotometer (PerkinElmer, LAMBDA™, Shelton, CT, USA) at 37 °C. Catalase activity was measured according the method previously described^57^. Glutathione peroxidase activity was measured by NADPH oxidation at 340nm^58^ and superoxide dismutase activity was assayed by the reduction of cytochrome C at 550nm^59^.

### Thiol content and carbonyl groups quantification

Total reduced thiols were determined using 5,5-dithionitrobenzoic acid (DTNB). Thiol residues react with DTNB, cleaving the disulfide bond to give 2-nitro-5-thiobenzoate (NTB^−^), which ionizes to the NTB^2−^ dianion in water at neutral and alkaline pH. NTB^2−^ was quantified in a spectrophotometer by measuring the absorbance at 412 nm and data was expressed as nmol of reduced DTNB/mg protein^60^. The proteins bound to carbonyl were evaluated based on the reaction with dinitrophenilhidrazine (DNPH), as previously described^61^. The carbonyl groups were measured at 370 nm.

### Thin-layer chromatography (TLC)

Liver total lipids were extracted by the method previously described^62^ with some modifications. After incubation in chloroform-methanol-water solution (2:1:0.8, v/v), samples were centrifuged (1500 × g for 20 min at 4 °C). Then, the chloroform was added to the collected supernatant. After centrifugation (1500 × g for 20 min) the ordic phase was removed and dried in nitrogen. The extracted lipids were analyzed by TLC for neutral lipids, using a DC Silicagel 60 plate (Merck Millipore, HE, Germany). The plates were submerged for 10 s in Charring solution (3% CuSO4 and 8% H3PO4 v/v), then they were dried and heated to 110 °C for 10 min. TLC plates were analyzed by densitometry (ImageQuant LAS 4000, GE Healthcare, Buckingham, Shire, UK). We analyzed free cholesterol, cholesterol ester, non-esterification fatty acid and triglycerides.

### H_2_O_2_ generation

Liver tissue was homogenized in a 50 mM sodium phosphate buffer, pH 7.2, containing 0.25 M sucrose, 0.5 mM dithiothreitol, 1 mM ethylene glycol tetra-acid (EGTA), 5 mg/mL aprotinin, and 34.8 mg/mL phenyl methane sulfonyl fluoride (PMSF). The homogenates were centrifuged at 3000 × g for 15 min at 4 °C. The membrane fraction was obtained by the resuspension of the pellet in assay buffer (0.5 mL 50 mM sodium phosphate buffer, pH 7.2, containing 0.25 M sucrose, 2 mM MgCl_2_, 5 mg/ml aprotinin and 34,8 mg/ml phenylmethanesulfonyl fluoride (PMSF)). To obtain the microsomal fraction, the supernatant was centrifuged at 100,000 × g for 35 min at 4 °C and the pellets were resuspended in assay buffer. Then, the supernatant was centrifuged at 100,000 × g for 35 min at 4 °C and the pellets were resuspended in assay buffer and were used for analyses. Both fractions were incubated in 150 mM sodium phosphate buffer (pH 7.4) containing SOD (100 U/ml; Sigma, USA), horseradish peroxidase (0.5 U/ml, Roche, Indianapolis, IN), Amplex red (50 µM; Molecular Probes, Eugene, OR), 1 mM EGTA in the presence or absence of 1 mM NADPH. The fluorescence was immediately measured in a microplate reader (Victor X4; PerkinElmer, Norwalk, CT) at 30 °C, using excitation at 530 nm and emission at 595 nm^63^.

### Transmission Electron Microscopy (TEM)

The fragments of liver were processed and analyzed qualitatively using a JEM1011 (JEOL) microscope. The fragments were washed in PBS and fixed for 24 h in a solution containing 2.5% glutaraldehyde in 0.1 M sodium cacodylate buffer (pH 7.2). Then, the samples were washed for 10 min in the same buffer. This wash step was repeated 3 times. The tissue was post-fixed for 1 h with a 1% osmium tetroxide (OsO_4_) solution in 0.1 M sodium cacodylate buffer (pH 7.2), dehydrated in an acetone series (30, 50, 70, 90 and 100%) and embedded in Poly/Bed(r) 812 resin (Ted Pella Inc, Redding, CA, USA). After polymerization, ultrathin sections were obtained and contrasted with uranyl acetate-lead citrate for ultrastructural observation. Were collected in 3 grids with 7 micrometers between them, acquired 10 images of random fields in each grid, quantified the total number of mitochondria and the number of injured mitochondria to obtain the percentage of injured mitochondria.

### Picrosirius red collagen staining

For the histological analysis, the liver was collected, fixed with 4% formalin, cleared and embedded in histological paraffin. The fixed samples were sectioned using a microtome in semi serials cuts 5 µm thick. After deparaffinization, the sections were rehydrated and incubated with 0.2% phosphomolybdic acid (Merk, Darmstadt, Germany) for 1 min and then incubated with picrosirius red solution for 90 min. After this, the sections were washed with 0.01 N of chloridric acid for 2 min and then in 70% alcohol for 45 seconds. The sections were then dehydrated with two successive washes of 5 min with 95% and 100% alcohol. After two washes of 100% xylol for 5 min, the blades were mounted and visualized in microscope (Axiovert 100-Carl Zeiss). The number of collagen fibers were quantified (15 fields for animal) using the Image Pro Plus® version 4.5 software (Media Cybernetics, Silver Spring, MD, USA).

### Data analysis

All data are presented as the mean ± standard error from mean (S.E.M.). Data sets were tested for normality using the Kolmogorov–Smirnov test, and the differences between control and HF offspring per each sex were analyzed by Mann-Whitney test or Student’s *t* test when appropriated. A value of p < 0.05 was considered statistically significant. Statistical analyses were performed in GraphPad Prism version 6.0 for Windows (GraphPad Software Inc., San Diego, CA, USA).

## Data Availability

The datasets generated during and/or analyzed during the current study are available from the corresponding author.

## References

[1] Kwon EJ, Kim YJ (2017). What is fetal programming?: a lifetime health is under the control of in utero health. Obstetrics & gynecology science.

[2] Elshenawy S, Simmons R (2016). Maternal obesity and prenatal programming. Mol Cell Endocrinol.

[3] Desai M, Jellyman JK, Ross MG (2015). Epigenomics, gestational programming and risk of metabolic syndrome. Int J Obes (Lond).

[4] Franco JG (2012). Maternal high-fat diet induces obesity and adrenal and thyroid dysfunction in male rat offspring at weaning. J Physiol.

[5] Oliveira LS (2016). Perinatal maternal high-fat diet promotes alterations in hepatic lipid metabolism and resistance to the hypolipidemic effect of fish oil in adolescent rat offspring. Molecular nutrition & food research.

[6] Franco JG (2016). Resveratrol treatment rescues hyperleptinemia and improves hypothalamic leptin signaling programmed by maternal high-fat diet in rats. Eur J Nutr.

[7] Almeida MM (2017). Perinatal maternal high-fat diet induces early obesity and sex-specific alterations of the endocannabinoid system in white and brown adipose tissue of weanling rat offspring. The British journal of nutrition.

[8] Dias-Rocha CP (2018). Maternal high-fat diet induces sex-specific endocannabinoid system changes in newborn rats and programs adiposity, energy expenditure and food preference in adulthood. The Journal of nutritional biochemistry.

[9] Ahima RS (2011). Digging deeper into obesity. The Journal of clinical investigation.

[10] Matias I, Di Marzo V (2007). Endocannabinoids and the control of energy balance. Trends Endocrinol Metab.

[11] Ruby MA (2011). Acute overactive endocannabinoid signaling induces glucose intolerance, hepatic steatosis, and novel cannabinoid receptor 1 responsive genes. PloS one.

[12] D’Addario C (2014). Endocannabinoid signaling and food addiction. Neurosci Biobehav Rev.

[13] Roche R (2006). Presence of the cannabinoid receptors, CB1 and CB2, in human omental and subcutaneous adipocytes. Histochemistry and cell biology.

[14] Nunez E (2004). Cannabinoid CB2 receptors are expressed by perivascular microglial cells in the human brain: an immunohistochemical study. Synapse.

[15] Wang M, Meng N, Chang Y, Tang W (2016). Endocannabinoids signaling: Molecular mechanisms of liver regulation and diseases. Frontiers in bioscience.

[16] Basu PP, Aloysius MM, Shah NJ, Brown RS (2014). Review article: the endocannabinoid system in liver disease, a potential therapeutic target. Alimentary pharmacology & therapeutics.

[17] Han KH (2009). CB1 and CB2 cannabinoid receptors differentially regulate the production of reactive oxygen species by macrophages. Cardiovascular research.

[18] Despres JP, Golay A, Sjostrom L (2005). & Rimonabant in Obesity-Lipids Study, G. Effects of rimonabant on metabolic risk factors in overweight patients with dyslipidemia. The New England journal of medicine.

[19] Hussien NI, El-Kerdasy HI, Ibrahim ME (2017). Protective effect of rimonabant, a canabinoid receptor 1 antagonist, on nonalcoholic fatty liver disease in a rat model through modulation of the hepatic expression of activin A and follistatin. Can J Physiol Pharmacol.

[20] Kasapoglu M, Ozben T (2001). Alterations of antioxidant enzymes and oxidative stress markers in aging. Experimental gerontology.

[21] Frankenfeld SP (2014). The anabolic androgenic steroid nandrolone decanoate disrupts redox homeostasis in liver, heart and kidney of male Wistar rats. PloS one.

[22] Biernacki M (2016). Crosstalk between liver antioxidant and the endocannabinoid systems after chronic administration of the FAAH inhibitor, URB597, to hypertensive rats. Toxicology and applied pharmacology.

[23] Lipina C, Hundal HS (2016). Modulation of cellular redox homeostasis by the endocannabinoid system. Open biology.

[24] Craft RM, Marusich JA, Wiley JL (2013). Sex differences in cannabinoid pharmacology: a reflection of differences in the endocannabinoid system?. Life Sci.

[25] Maia J., Almada M., Silva A., Correia-da-Silva G., Teixeira N., Sá S.I., Fonseca B.M. (2017). The endocannabinoid system expression in the female reproductive tract is modulated by estrogen. The Journal of Steroid Biochemistry and Molecular Biology.

[26] Tellechea ML, Mensegue MF, Pirola CJ (2017). The Association between High Fat Diet around Gestation and Metabolic Syndrome-related Phenotypes in Rats: A Systematic Review and Meta-Analysis. Sci Rep.

[27] Osei-Hyiaman D (2005). Endocannabinoid activation at hepatic CB1 receptors stimulates fatty acid synthesis and contributes to diet-induced obesity. The Journal of clinical investigation.

[28] Teixeira-Clerc F (2006). CB1 cannabinoid receptor antagonism: a new strategy for the treatment of liver fibrosis. Nature medicine.

[29] De Gottardi A, Spahr L, Ravier-Dall’Antonia F, Hadengue A (2010). Cannabinoid receptor 1 and 2 agonists increase lipid accumulation in hepatocytes. Liver international: official journal of the International Association for the Study of the Liver.

[30] Soethoudt M (2017). Cannabinoid CB2 receptor ligand profiling reveals biased signalling and off-target activity. Nat Commun.

[31] Rossi F (2012). Cannabinoid receptor type 2 functional variant influences liver damage in children with non-alcoholic fatty liver disease. PLoS One.

[32] Auguet T (2014). Endocannabinoid receptors gene expression in morbidly obese women with nonalcoholic fatty liver disease. Biomed Res Int.

[33] Rodriguez A, Ezquerro S, Mendez-Gimenez L, Becerril S, Fruhbeck G (2015). Revisiting the adipocyte: a model for integration of cytokine signaling in the regulation of energy metabolism. Am J Physiol Endocrinol Metab.

[34] Julien B (2005). Antifibrogenic role of the cannabinoid receptor CB2 in the liver. Gastroenterology.

[35] Deveaux V (2009). Cannabinoid CB2 receptor potentiates obesity-associated inflammation, insulin resistance and hepatic steatosis. PLoS One.

[36] Anstee QM, Goldin RD (2006). Mouse models in non-alcoholic fatty liver disease and steatohepatitis research. International journal of experimental pathology.

[37] Bradbury MW, Berk PD (2004). Lipid metabolism in hepatic steatosis. Clinics in liver disease.

[38] Armitage JA, Taylor PD, Poston L (2005). Experimental models of developmental programming: consequences of exposure to an energy rich diet during development. The Journal of physiology.

[39] McCurdy CE (2009). Maternal high-fat diet triggers lipotoxicity in the fetal livers of nonhuman primates. The Journal of clinical investigation.

[40] Mouralidarane A (2015). Maternal obesity programs offspring non-alcoholic fatty liver disease through disruption of 24-h rhythms in mice. Int J Obes (Lond).

[41] Mouralidarane A (2013). Maternal obesity programs offspring nonalcoholic fatty liver disease by innate immune dysfunction in mice. Hepatology.

[42] Aiken CE, Ozanne SE (2013). Sex differences in developmental programming models. Reproduction.

[43] Lecoutre S (2016). Depot- and sex-specific effects of maternal obesity in offspring’s adipose tissue. The Journal of endocrinology.

[44] Proto MC (2012). Interaction of endocannabinoid system and steroid hormones in the control of colon cancer cell growth. Journal of cellular physiology.

[45] Grimaldi P (2012). The faah gene is the first direct target of estrogen in the testis: role of histone demethylase LSD1. Cell Mol Life Sci.

[46] Ramirez-Lopez MT (2016). Exposure to a Highly Caloric Palatable Diet during the Perinatal Period Affects the Expression of the Endogenous Cannabinoid System in the Brain, Liver and Adipose Tissue of Adult Rat Offspring. PLoS One.

[47] Ramirez-Lopez MT (2016). Long-Term Effects of Prenatal Exposure to Undernutrition on Cannabinoid Receptor-Related Behaviors: Sex and Tissue-Specific Alterations in the mRNA Expression of Cannabinoid Receptors and Lipid Metabolic Regulators. Frontiers in behavioral neuroscience.

[48] Gandhi K (2018). Effect of maternal high-fat diet on key components of the placental and hepatic endocannabinoid system. Am J Physiol Endocrinol Metab.

[49] Cao Z (2013). Monoacylglycerol lipase controls endocannabinoid and eicosanoid signaling and hepatic injury in mice. Gastroenterology.

[50] Ahmadi A, Shadboorestan A (2016). Oxidative stress and cancer; the role of hesperidin, a citrus natural bioflavonoid, as a cancer chemoprotective agent. Nutrition and cancer.

[51] Jia J (2014). Anandamide protects HT22 cells exposed to hydrogen peroxide by inhibiting CB1 receptor-mediated type 2 NADPH oxidase. Oxidative medicine and cellular longevity.

[52] Jorgacevic B (2015). Rimonabant Improves Oxidative/Nitrosative Stress in Mice with Nonalcoholic Fatty Liver Disease. Oxidative medicine and cellular longevity.

[53] Batkai S (2007). Cannabinoid-2 receptor mediates protection against hepatic ischemia/reperfusion injury. FASEB journal: official publication of the Federation of American Societies for Experimental Biology.

[54] Pacher P, Mechoulam R (2011). Is lipid signaling through cannabinoid 2 receptors part of a protective system?. Progress in lipid research.

[55] World Medical A, American Physiological S (2002). Guiding principles for research involving animals and human beings. Am J Physiol Regul Integr Comp Physiol.

[56] Fischbeck KL, Rasmussen KM (1987). Effect of repeated reproductive cycles on maternal nutritional status, lactational performance and litter growth in ad libitum-fed and chronically food-restricted rats. J Nutr.

[57] Aebi H (1984). Catalase *in vitro*. Methods in enzymology.

[58] Flohe L, Gunzler WA (1984). Assays of glutathione peroxidase. Methods in enzymology.

[59] Crapo JD, McCord JM, Fridovich I (1978). Preparation and assay of superoxide dismutases. Methods in enzymology.

[60] Fortunato RS (2013). Sexual dimorphism of thyroid reactive oxygen species production due to higher NADPH oxidase 4 expression in female thyroid glands. Thyroid: official journal of the American Thyroid Association.

[61] Levine RL (1990). Determination of carbonyl content in oxidatively modified proteins. Methods in enzymology.

[62] Bligh EG, Dyer WJ (1959). A rapid method of total lipid extraction and purification. Canadian journal of biochemistry and physiology.

[63] Fortunato RS (2010). Functional consequences of dual oxidase-thyroperoxidase interaction at the plasma membrane. The Journal of clinical endocrinology and metabolism.

